# A Hybrid Network Integrating 1DCNN, GRU, and ISENet for Pulse Signal Pattern Analysis

**DOI:** 10.1155/jp/2147025

**Published:** 2026-09-08

**Authors:** Nan Li, Jiarui Yu, Yuping Zhao, Lifeng Dong

**Affiliations:** ^1^ China Academy of Chinese Medical Sciences, Beijing, China, cacms.ac.cn; ^2^ China Academy of Chinese Medical Sciences, Wangjing Hospital, Beijing, China, cacms.ac.cn; ^3^ Institute of Automation Chinese Academy of Sciences, Beijing, China

**Keywords:** gated recurrent unit, improved squeeze and excitation network, one-dimensional convolutional neural network, pregnancy pulse

## Abstract

**Objective:**

Pulse diagnosis holds significant importance in traditional Chinese medicine (TCM). The accurate identification of pregnancy pulse patterns is crucial in TCM diagnosis, as it provides valuable insights into a patient′s health status and enables the development of personalized treatment strategies. However, the complexity and variability of pulse characteristics across different stages of pregnancy pose a significant challenge for TCM practitioners.

**Methods:**

To address the issue, a novel one‐dimensional convolutional neural network (1DCNN) integrated with a gated recurrent unit (GRU) and an improved squeeze‐and‐excitation network (ISENet), named 1DCNN–GRU–ISENet, is proposed to identify the pregnancy pulse of three stages. Specifically, the 1DCNN is employed to construct parallel convolutional branches and series‐stacked blocks. This architecture not only functions as a residual network but also enhances the network′s depth, enabling effective extraction of pulse features. By incorporating this structure, the model is capable of capturing more intricate and subtle characteristics of the pulse within different frequency ranges, leading to improved feature representation and enhanced performance. Simultaneously, the integration of GRUs facilitates comprehensive learning of pulse temporal features. Moreover, the ISENet assigns varying weights to different feature components based on pulse trends, effectively focusing on and highlighting crucial spatial features.

**Results:**

Through rigorous evaluation with a locally collected research dataset, our network achieves accuracy rates of 90.8%, 91.6%, and 91.5% in identifying pregnancy pulse patterns for the three stages, respectively. Additionally, our model demonstrates good performance across various metrics, including average precision, sensitivity, F1 score, and AUC, with scores of 91.7%, 91.5%, 0.916, and 97.1%, respectively.

**Conclusion:**

These results underscore the effectiveness of our proposed network in accurately classifying pulse patterns and showcase its potential for practical application in assisting with auxiliary diagnosis in the context of TCM pulse evaluation.

## 1. Introduction

Pulse diagnosis, a cornerstone of traditional Chinese medicine (TCM)′s four diagnostic methods, is widely employed in clinical diagnosis [1–3]. TCM physician uses the fingers to feel the pulse at the radial artery, extracting pulse information and utilizing it as a crucial basis for clinical differentiation and diagnosis [4–6]. Pulse analysis plays a pivotal role in understanding the physiological conditions of individuals, including during pregnancy, a process marked by significant physiological changes. From the perspective of TCM, pregnancy is divided into three stages: early (E), middle (M) and late (L), each with distinct pulse patterns [7]. The pulse at different stages of pregnancy represents the body′s adaptive response to increased metabolic demands and changes in blood volume [8, 9]. Studying these changes can provide a deeper understanding of the physiological adaptations required to support fetal growth and help evaluate the cardiovascular health status of mothers [10, 11].

Modern research indicates that pulse wave is a one‐dimensional waveform signal generated by the pulsation of the heart causing blood to flow in the arteries [12, 13]. Identifying and analyzing pregnancy pulse waves can be used to monitor physiological changes induced by pregnancy. However, in practical applications, accurately estimating waveform metrics is often challenging, especially for nonprofessionals.

To address this, researchers have utilized a range of classical analysis techniques. Wang′s decision tree–based method quantifies pulse wave intensity [14], whereas Su et al. [15] explore the impact of pregnancy trimester specific cardiovascular changes on photoplethysmography (PPG) signals, peripheral blood pressure (BP), and heart rate (HR). Chen et al. [16] utilized a BP network for the classification of pregnancy pulse, achieving an average accuracy of 84.7% and an AUC of 91%. Lu et al. [17] extracted time domain and wavelet domain features from pulse data. They employed various machine learning algorithms, including adaptive boosting (AdaBoost), BPNN, SVM, and PNN to identify pregnancy pulse. The AdaBoost classifier achieved an accuracy of 86.58% and an AUC of 94%, underscoring the viability of using machine learning for pregnancy pulse detection. Li et al. [18] developed CNN and GRU networks for discriminating pulses at different stages of pregnancy, yielding impressive classification results and performance metrics. Wang et al. [19] employed harmonic characteristics to classify pregnancy pulses across three trimesters, achieving an average accuracy of 89.7%. Li et al. [20] proposed new pulse wave pattern classification criteria and a matching CNN model. The optimized network model achieved a recognition rate of 89% for pulse waves. However, most previous studies required substantial manual feature extraction to differentiate pulse signals, overlooking the temporal and spatial relationships between pulse waves. Human and environment‐related interferences often undermine the stability of their methods, and the small test datasets used for evaluation limit the generalization of results.

The integration of AI with stage‐specific maternal health monitoring has recently attracted growing interest. Systems for trimester‐aware health recommendations [21] and AI‐assisted nutritional guidance during pregnancy [22] demonstrate how physiological stage information can drive personalized interventions. Complementing this, federated learning frameworks [23] offer privacy‐preserving approaches to multi‐institutional model training, a critical consideration for generalizing pulse diagnosis models across diverse clinical settings. More broadly, precision medicine applications leveraging molecular and physiological AI [24] underline the expanding role of deep learning in clinical decision support—a paradigm our work advances in the context of TCM pulse diagnosis.

To better integrate spatial and temporal information for physiological data identification, CNNs and recurrent neural networks (RNNs) have been introduced [25, 26]. CNNs, especially 1DCNN, can directly process pulse wave signals by automatically extracting relevant features through one‐dimensional convolutional kernels, reducing information extraction redundancy [27, 28]. Traditional RNNs face gradient explosion and vanishing problems, which can be alleviated by GRU and long short‐term memory (LSTM) units [29, 30]. The attention mechanism, a nonlocal information fusion technique, has also been incorporated into CNN and GRU modules to optimize information weight allocation [31, 32]. Currently, the majority of existing literature primarily focuses on analyzing the morphological features of pulse waves, and research on identifying pulse wave patterns for different stages of pregnancy is limited. In fact, understanding the pulse patterns at different stages of pregnancy holds tremendous potential in gaining valuable insights into the overall health aspects of pregnant women. As far as we know, the identification of pregnancy pulse remains an ongoing and challenging problem that warrants further exploration.

Motivated by the previous works [16, 18, 29], the identification model of pregnancy pulse in each stage was proposed by the combination of 1DCNN, GRU, and ISENet. In the proposed method, we constructed parallel convolutional branches and series stacked blocks. This architecture empowers the model to adeptly capture subtle pulse characteristics across diverse frequency ranges, resulting in improved feature representation and enhanced performance. Moreover, it provides the flexibility to fine‐tune convolutional scales and depths, enabling the attainment of optimal outcomes. The integration of GRU units facilitates comprehensive learning of pulse temporal sequences. Meanwhile, ISENet assigns different weights to various feature components based on the pulse trends, effectively focusing and highlighting key spatial features, enhancing the network′s generalization and robustness. To the best of our knowledge, the research on pulse of pregnancy identification problem is still open and remains challenging.

Our original contributions of the work are as follows: (1) Two local feature‐learning blocks (Blocks A and B) are designed for multifrequency feature extraction; (2) the model adopts parallel convolution branches and concatenated stacking blocks to enhance network depth and feature extraction; and (3) GRU units are introduced to capture long‐term temporal dependencies from pulse sequences. An improved SENet (ISENet) is proposed with four specific structural improvements over standard SENet, enabling spatiotemporal feature recalibration.

The remainder of the study is organized as follows. The materials and methods are introduced in Section 2. Section 3 introduces the experiment preparation work. In Section 4, the results and discussion are given. Finally, we briefly draw conclusions and give directions for future works in Section 5.

## 2. Methods

### 2.1. Subjects Information

**Figure 1:**
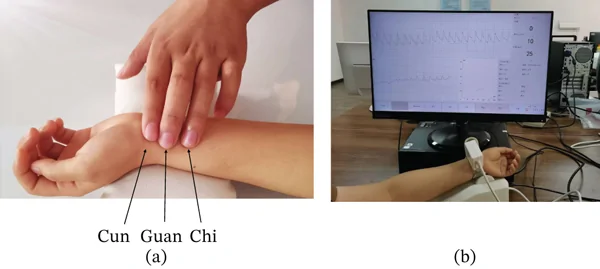
(a,b) The pulse acquisition.

Pulse waveform measurement and data collection were completed at the Obstetrics and Gynecology Hospital of Fudan University. We measured and recorded pulse waveform signals from 184 pregnant women, including 67 in the early stage, 61 in the middle stage, and 56 in the late stage of pregnancy. The characteristics of the subjects are summarized in Table 1. All subjects met the diagnostic criteria and inclusion criteria. Each study participant obtained written informed consent (approved by the Ethics Committee of China Academy of Chinese Medical Sciences (Reference Number: CACMSIRB2020‐005)), and all experiments were conducted in accordance with relevant guidelines and regulations. The typical waveforms acquisition is shown in Figure 1 [33].

**Table 1:** Baseline characteristics of subjects in the three stages.

	E stage	M stage	L stage	*p*
Age (year)	26 ± 5	30 ± 3	32 ± 4	n.s.
Gestational age (week)	8–12	24–28	32–36	—
Body height (cm)	161.4 ± 4.2	160.7 ± 5.4	161.5 ± 6.2	n.s.
Body weight (kg)	55.7 ± 8.5	57.4 ± 6.4	68.9 ± 7.8	n.s.
BMI (kg/m^2^)	22.1 ± 2.7 ^∗^^•^	25.1 ± 3.8^◊•^	28.2 ± 3.6^◊^ ^∗^	0.00
SBP (mmHg)	108 ± 17	103 ± 11	105 ± 13	0.33
DBP (mmHg)	70 ± 11 ^∗^^•^	65 ± 9^◊^	62 ± 12^◊^	0.01

*Note:* Values are means ± SD. Compared with the first trimester, ^◊^ *p* < 0.05; compared with the second trimester,  ^∗^ *p* < 0.05; and compared with the third trimester, ^•^ *p* < 0.05 [7, 9].

Abbreviations: BMI, body mass index; DBP, diastolic blood pressure; SBP, systolic blood pressure.

**Figure 2:**
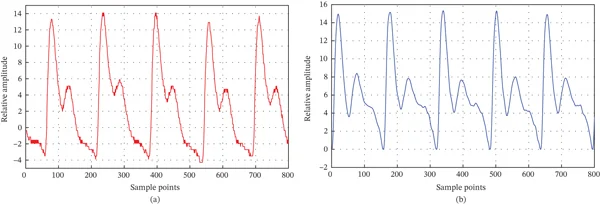
(a,b) The preprocessed pulse signal.

Due to factors such as the inherent movement of limbs and sensors, as well as interference from breathing and power frequency, the purity of the pulse wave is prone to contamination. Therefore, the wavelet transform is used to preprocess the collected pregnancy pulse signals [34, 35]. Figure 2 shows the preprocessed pulse signal. Furthermore, data overlap sampling and sliding segmentation methods were used to segment the pulse wave sample data. Data augmentation primarily utilized overlapping sampling and sliding segmentation methods. Specifically, a fixed‐length window was selected from the preprocessed pregnancy pulse signals for segmentation. By setting an overlap rate (50%), each new window moved with a portion overlapping the previous one. This approach ensures that more samples are extracted from the same signal, thereby increasing the diversity of the training data. The method is as follows:

(1) N=L−1D+1,

where *L* is the length of pulse sequence, and *D* is the step size.

### 2.2. The Architecture of Hybrid Model

**Figure 3:**
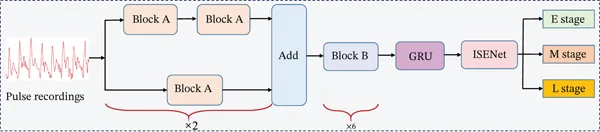
The architecture of proposed model.

As shown in Figure 3, the hybrid model consists of three parts: 1DCNN, GRU, and ISENet.

#### 2.2.1. 1DCNN Units

**Figure 4:**
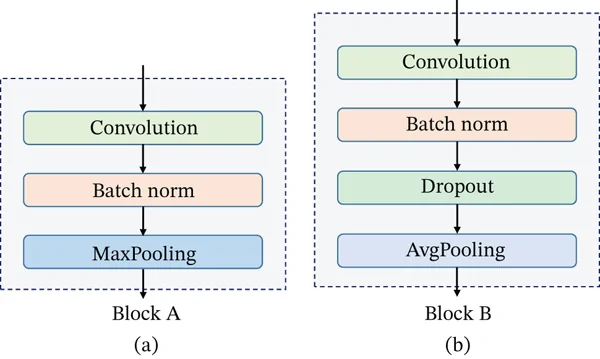
(a,b) The architecture of Block A and B.

Since the collected pulse signals are all time series, 1DCNN networks are designed in this paper to effectively extract the key information of pregnancy pulse signal. The framework of 1DCNNs can be divided into two parts as shown in Figure 4. Firstly, the forward convolutional unit consists of two parallel Block A branches, each independently extracting pulse features at different frequency bands. Specifically, the two Block A branches receive the same input and produce feature maps that are subsequently concatenated along the channel dimension, thereby enabling multiscale frequency‐domain feature fusion. After each Block A, the feature maps are fed into a batch normalization (BN) layer and a MaxPooling layer to reduce dimensionality and suppress redundant information. This not only helps to compress the information but also reduces the overall computational load.

Similar to Block A, Block B consists of a convolutional layer, a BN operation, a dropout layer, and an Avgpooling layer. The backbone of the hybrid network consists of six Block B, which achieve secondary extraction of the most relevant and prominent features of the input pulse wave features and construct an activation map. Pulse feature vector u⊆k^t×f^ is generated by passing the input time steps features to the convolution layer, where *t* and *f* are the value of time step and features, respectively. The mathematical operation of the 1DCNN layer is

(2) yjl=∑i=1Mxil⊗kijl+bji,

where *y* is the vector obtained after calculating the *j*th convolution of layer *l*; *M* is the number of input feature vectors; $x_{i}^{l}$ is the *i*th input feature vector of layer *l*; ⊗ represents related operations; $k_{ij}^{l}$ is the *j*th convolution kernel that convolves layer *l* with the *i* input feature vector; $b_{j}^{i}$ is the *j*th bias vector of layer *l*.

Considering the convergence speed and overfitting issues, this article adopts the ReLU as the hidden layer activation function, which can improve the sparsity of the network and effectively prevent overfitting problems. After ReLU activation, in order to increase the sparsity of the model and improve the speed of network training, the pooling layer is used to reduce the feature dimension learned by the convolutional layer. The 1DCNN kernel slides on the input pulse sequence, performs convolution operations, and extracts the features of the original pulse data through nonlinear activation, weight, and bias, ultimately obtaining a flat subsequent data point containing multiple features.

#### 2.2.2. GRU Unit

**Figure 5:**
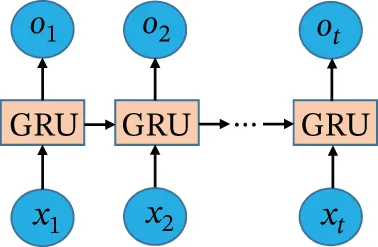
GRU network model.

**Figure 6:**
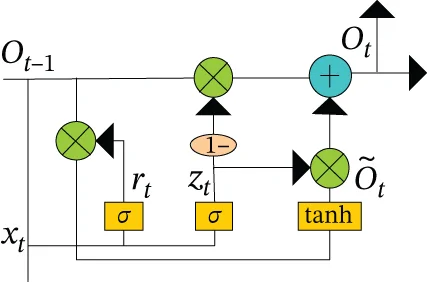
The internal structure of GRU.

The GRU unit addresses the challenges of gradient vanishing and exploding associated with long‐term dependencies in traditional RNNs. Unlike LSTM networks, GRU employs a simpler architecture with only two critical components: the update gate and the reset gate units. The specific structure of GRU is shown in Figures 5 and 6, respectively. The reset gate determines how incoming data should blend with the previous memory, whereas the update gate decides how much of the prior memory should be preserved at the current time step. In our proposed model, the GRU unit is used as the middle part of the hybrid model. The output pulse feature vector of the 1DCNN unit serves as the input of the GRU unit, where the global long‐term dependence of the pulse sequence is captured. The GRU model is presented in the form:

(3) rt=gt⊙r~t+1−gt⊙rt−1,

(4) r~t=δNgXt+Ohgt⊙rt−1+bh,

where *r*_*t*_ and *r*_*t*−1_ are the current and previous states of GRU unit, respectively. *g*_*t*_ and *g*_*r*_ are update doors and rest doors, respectively. *N*_*g*_ and *O*_*h*_ are weight matrices, *δ* is the activation function, and *b*_*h*_ is the bias. The process for updating and resetting doors is as follows:

(5) gt=δNgXt+Ogrt−1+bg,

(6) gr=δNrXt+Orrt−1+br.

In our experiment, the input vector is the output of 1DCNN, which is u, so the GRU unit becomes as follows. The current situation of GRU can be calculated using Equation (7)

(7) r~t=δNgut+Ohgr⊙rt−1+bh.

#### 2.2.3. Squeeze‐and‐Excitation Network

**Figure 7:**
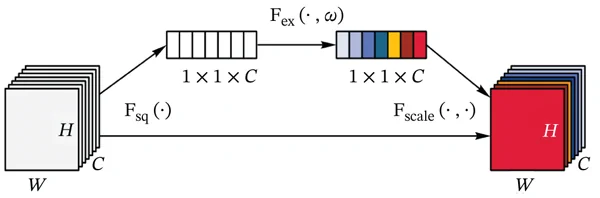
The structure of SENet.

The squeeze‐and‐excitation operation was introduced by Hu et al. [36, 37] in 2018. As shown in Figure 7, the SE network consists mainly of three components: the squeeze operation, the excitation operation, and the feature map rescaling operation. These components work together to achieve the adaptive recalibration of relationships between pulse waves. Initially, the squeeze operation is performed using global average pooling to extract global information from each feature map channel, as indicated by Equation (8)

(8) Zc=FsqUc=1H×W∑m=1H∑n=1WUcm,n,

where *F*_*s* *q*_ represents the squeeze operation; *U*_*c*_ denotes the tensor of feature maps; and *c* stands for the channel index.

Subsequently, the feature vector *Z* obtained from the squeeze operation is fed into the excitation operation, as shown in Equation (9)

(9) S=FexZ,w=σgZ,w=σw2δw1Z,

where *F*_*e* *x*_ represents the excitation operation; *w*_1_ and *w*_2_ are the weight parameters for dimension reduction and dimension augmentation, respectively, which are associated with fully connected (FC) layers or convolutional layers; *σ* signifies the sigmoid function; *δ* stands for the ReLU activation function. Finally, the pulse channel weight vector *S* is element‐wise multiplied with *U* to obtain the rescaled feature maps. These rescaled feature maps are then passed to the next convolutional layer for further processing.

(10) X~c=FscaleUc,Sc=UcSc.

**Figure 8:**
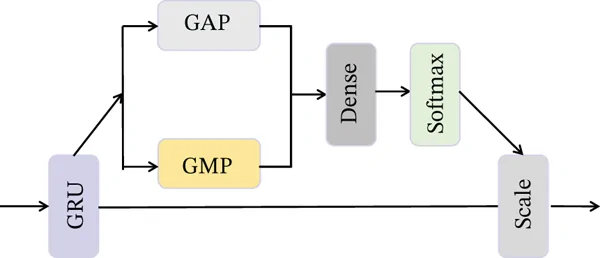
The structure of modified SENet.

In order to enhance the extraction of spatiotemporal features between pulse waves and allocate varying importance weights to pulse wave feature mappings, an improved version of the SENet (ISENet) has been proposed, as illustrated in Figure 8. Different from single GAP squeeze in standard SENet, ISENet adopts dual‐path parallel pooling of GAP and GMP to simultaneously extract global average amplitude and peak pulse features, and fuses two statistics via concat operation before dense mapping.

ISENet introduces improvements to each of the above steps across four independent dimensions:

1. GRU‐based temporal encoding of pulse wave trends: In the standard SENet, the excitation is performed by two FC layers with a bottleneck ratio *r*, applied independently to the pooled channel vector. ISENet introduces a GRU layer prior to squeezing, which models temporal dynamics across the pulse wave sequence {x_1_, x_2_, ⋯x_T_}. The GRU output encodes cross‐time step pulse trend information. It is then concatenated with the spatial descriptor from GAP to form a spatiotemporal joint representation:
(11) z∗=ConcatFsqU,hT.

This means the channel attention computed by ISENet acts on GRU hidden‐state channels, each of which encodes a distinct temporal dependency pattern across the pulse sequence. The effect is to reweight temporal feature channels according to their diagnostic relevance, enabling the model to adaptively suppress irrelevant temporal patterns and amplify those corresponding to stage discriminative waveform components. Furthermore, SENet′s *z* is a pure spatial statistic; ISENet′s *z*∗ is a joint spatiotemporal representation that can distinguish pulse features with similar morphology but different temporal progressions.

2. Softmax replaces sigmoid: Softmax generates a normalized probability distribution, which means that increasing the weight of one channel will inevitably suppress other channels, resulting in sparse attention allocation. This is critical for pulse wave analysis, where only a few feature channels (e.g., main wave and dicrotic wave) are salient at any given moment.
3. Dense weight correlation mapping: Standard SENet employs a bottleneck FC structure, which necessarily discards interchannel relational information during compression. However, ISENet replaces this with a direct dense mapping. This operation directly projects the spatiotemporal joint feature *z*∗ to C‐dimensional channel weight logits, preserving complete cross‐channel correlation information and avoiding information loss from the SENet bottleneck.
4. Parallel dual‐path squeeze: In addition to the above three improvements, ISENet employs a parallel dual‐path squeeze that combines global average pooling and global max pooling. The two resulting channel descriptors are concatenated and then fused via a 1 × 1 convolution. This enriches the channel statistics used for excitation, allowing the network to capture both mean‐level and peak‐level activations that characterize the dominant pulse morphology at each stage of pregnancy.

Compared with standard SENet that only extracts static spatial channel statistics, the proposed ISENet constructs spatiotemporal joint features and introduces competitive channel weighting, which is specially designed to capture time‐varying pulse waveform characteristics across different pregnancy stages.

#### 2.2.4. The Recognition Algorithm Flow

Initially, the pulse data undergoes segmentation into Nseg subsamples, each containing Nsub = Ninput/Nseg data points. Subsequently, two parallel Block A branches perform multiscale frequency feature extraction, with each branch independently applying convolution at different receptive‐field scales; the outputs are concatenated to form the initial feature representation. Six sequentially stacked Block B units then serve as the deep feature extraction backbone, progressively refining and abstracting the local pulse features. Max pooling and average pooling operations are subsequently applied. The convolution output feature set is then fed into a GRU layer for temporal sequence modeling. The GRU module facilitates the incorporation of pulse temporal sequence information by connecting the forward and backward hidden states. The GRU state vector′s output is weighted by ISENet, producing a set of weighted intermediate states as candidate outputs. Weight values between each state are determined based on the correlation between the output state and intermediate state. These weight values are then normalized, aggregated, and summed. This process yields a final representation vector that consolidates all input pulse feature information. The softmax function is employed to generate a probability vector representing different stages of pregnancy, ultimately achieving pregnancy pulse classification. The optimal parameters have been given through numerous experiments, as summarized in Table 2.

**Algorithm: 1DCNN–GRU–ISENet training and inference procedure.**
Input: Raw pulse signals S from N subjects; Labels Y; parameters (lr, batch_size, epochs)
Output: Trained model *Θ* ∗; Performance metrics on test set
Step 1: Preprocessing
 For each subject s_i_ ∈ S:
  Apply wavelet denoising (PyWavelets) to remove motion/breathing artifacts
Step 2: Patient‐Wise Split
 Randomly assign 148 subjects to Training Set T, 36 subjects to Test Set V
 Partition T into 5 folds for subject‐wise cross‐validation
 Step 3: Sliding Window Segmentation
 For each signal: apply sliding window (N_input=748, overlap=50%) → segments
 Step 4: Cross‐Validation Loop
 For fold k = 1 to 5:
  For epoch e = 1 to max_epochs:
   For each mini‐batch B from training fold:
    Forward pass:
    F_local_ ← 1DCNN(B)        // Block A × 2 (parallel) → Block B × 6
    H_T_ ← GRU(F_local_)       // Temporal sequence encoding
    z∗ ← Concat[Fsq(F_local_), H_T_]    // Spatiotemporal representation
    w ← Softmax(Wd · z∗ + b_d_)    // Dense channel attention weights
    F_att_ ← w ⊙ F_local_         // Weighted feature maps
    p ← Softmax(FC(F_att_))       // Class probability vector
    L ← CrossEntropy(p, y)      // Loss computation
    Backward pass:
    *Θ* ← Adam(*Θ*, ∇L, lr=0.005)    // Parameter update
   If validation loss does not improve for 50 epochs: break (early stopping)
   Evaluate fold k metrics: Acc_k_, Prec_k_, Sens_k_, Spec_k,_ F1_k_, AUC_k_
 Step 5: Final Evaluation
 Train final model *Θ* ∗ on full Training Set T with optimal hyperparameters
 Evaluate *Θ* ∗ on held‐out Test Set V → Report final metrics
 Report: mean ± SD across 5 folds for all metrics

**Table 2:** The structure of the proposed network.

Block	Layers	Filters	Length	Stride
	Input	—	748 × 1	1
A	CNN	32	9 × 1	1
A	BN	—	—	—
A	Max pooling	—	—	—
B	CNN 1–2	64	3 × 1	1
B	CNN 1–2	128	3 × 1	1
B	CNN 1–2	256	3 × 1	1
B	BN	—	—	—
B	Dropout	—	—	—
B	Max pooling	—	—	—
C	GRU	—	1 × 126	1
C	ISENet	—	1 × 126	1
C	Dense	—	32	1
C	Softmax	—	3	1

## 3. Experiments

### 3.1. Setup

After undergoing data augmentation and screening, a total of 17,294 pulse wave samples were generated from 184 subjects. Among these samples, there were 6298 samples from the early stage, 5344 samples from the middle stage, and 5652 samples from the late stage. To prevent data leakage caused by overlapping windows from the same patient appearing in both training and test sets, we adopted a strict patient‐wise (subject‐independent) split strategy. Specifically, among the 184 enrolled subjects, we randomly assigned 148 subjects to the training set and 36 subjects to the test set, ensuring no pulse windows from the same individual exist in both partitions. All augmented samples derived from a given subject were assigned entirely to either the training set or the test set, ensuring that no temporal segments from the same individual appeared in both partitions. Consequently, the test set only consists of windows extracted from reserved unseen subjects that were never involved in the training phase. This approach strictly conforms to the independent and identically distributed assumption for the test set and provides an unbiased estimation of the model′s generalization performance on completely new individuals. Furthermore, to provide a more statistically robust evaluation, we additionally performed a fivefold subject‐wise cross‐validation, in which the training set was partitioned into five folds and the entire pipeline was repeated five times. The mean and standard deviation of performance metrics across folds are reported in the revised Table 3.

**Table 3:** The performance of the proposed network.

Classes	Accuracy (%)	Precision (%)	Sensitivity (%)	Specificity (%)	F1
E stage	90.8 ± 0.41	91.6 ± 0.35	90.2 ± 0.33	91.5 ± 0.29	0.909
M stage	91.6 ± 0.25	92.3 ± 0.17	92.4 ± 0.56	91.2 ± 0.47	0.923
L stage	91.5 ± 0.38	91.4 ± 0.64	91.9 ± 0.72	90.7 ± 0.62	0.916

We evaluated the performance on a validation set and chose the learning rate that minimized the loss function. The learning rate was set to 0.005, as it provided the best balance between convergence speed and accuracy. Batch sizes of 16, 32, 64, and 128 were considered. A batch size of 32 was selected because it provided the best trade‐off between computational efficiency and model performance. A dropout rate of 0.2 was selected as it provided the best improvement in generalization without hindering training performance.

The experimental scheme is implemented by Python 3.10. The training and testing of this experiment were conducted on a Linux server equipped with an NVIDIA GeForce RTX 4090 GPU and 64 GB RAM. The deep learning framework used was pyTorch 1.13, running under CUDA 11.7.

### 3.2. Performance Evaluation

The performance of the proposed hybrid network is evaluated using the most popular four metrics: accuracy, specificity, precision, and F1 score, which are defined in (12), (13), (14), and (15), where TP, TN, FP, and FN are true positive, true negative, false positive, and false negative, respectively.

(12) Accuracy=TP+TNTP+TN+FP+FN,

(13) Sensitivity=TPFN+TP,

(14) Precision=TPTP+FP,

(15) F1 score=2Precision⋅recallPrecision+recall,

where TP denotes true positives, TN is true negatives, FP represents false positives, and FN indicates false negatives. A higher value for each metric indicates better classification performance. Accuracy provides an overall classification performance measure; precision and sensitivity capture the false‐alarm/missed detection trade‐off; F1‐score provides the harmonic mean particularly useful when class sizes differ. For fairness, the training was repeated five times for each trial on the samples and their average was taken as the final result.

The receiver operating characteristic curve (ROC) is a comprehensive representative of the detection accuracy, which reflects the relationship between the true rate and the false positive rate. The ROC curve also shows relationship between sensitivity and specificity. Area under the curve (AUC) is defined as the area under the ROC curve. AUC is an evaluation index to evaluate the merits of a classification model, indicating the probability that the predicted positive cases are ranked ahead of the negative cases.

(16) True positive rate=TPTP+FN×100%,

(17) False positive rate=FPFP+TN×100%.

## 4. Results and Discussion

In this section, we start by showcasing the recognition performance of the proposed model on pulse waves. Subsequently, we delve into the study and discussion of several pivotal parameters that affect the performance of the model. Afterward, we proceed with ablative experiments to analyze the influence of various modules on the model. Finally, we compare the model we put forward with several other learning methods and thoroughly discuss the results of this comparison.

### 4.1. Results

In this section, pregnancy pulse waves are classified using the proposed hybrid model and its efficiency and performance in performing feature extraction and classification tasks are investigated.

**Figure 9:**
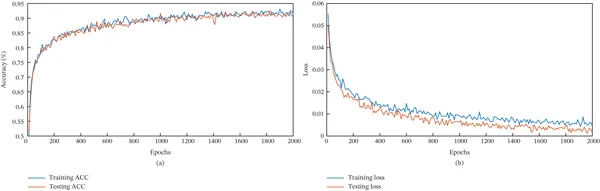
The accuracy and loss curves of the proposed model for pulse detection. (a) Accuracy curve. (b) Loss curve.

As depicted in Figure 9, the accuracy of pulse wave identification and the corresponding loss values stabilize around the 2000 iteration. Our experimental results demonstrate strong performance, with an average training accuracy of 91.3% and a loss of 0.15 over 2000 epochs for the 1DCNN–GRU–ISENet model. As the training accuracy reaches its peak and remains steady, the validation accuracy exhibits some fluctuations before eventually stabilizing. This phenomenon signifies that the network has achieved excellent predictive performance once the validation accuracy ceases to improve during training. Furthermore, our model excels in classifying pulse waves associated with different pregnancy stages. Specifically, we achieved an accuracy rate of 90.8% for the E stage, 91.6% for the M stage, and 91.5% for the L stage, underscoring the reliability and practicality of the proposed 1DCNN–GRU–ISENet network. In addition to high accuracy rates, our hybrid network also demonstrates impressive performance metrics for pregnancy pulse wave classification, including precision (91.7%), sensitivity (91.5%), specificity (91.1%), and an F1 score of 0.911. Li et al. [38] proposed a hybrid deep learning framework that uses 1DCNN, GRU, and attention mechanisms for pregnancy pulse classification. In this study, we advanced the research by introducing an ISENet to enhance the network′s ability to capture spatial features. Although Li et al. [38] achieved high accuracy in pregnancy pulse classification, this study optimized the architecture and achieved better performance on multiple evaluation metrics, such as improved average precision, sensitivity, and F1 score. The specific performance indicators for the three stages are provided in Table 3.

### 4.2. Parameter Analysis

#### 4.2.1. Pulse Wave Signal Length

The length of the pulse wave signal is an important influencing factor that may have significant impact on the classification results. To understand this effect more comprehensively, we conducted a series of experiments to compare the lengths of the pulse wave signal in the classification task. Initially, we selected four different lengths of pulse wave signals, namely 256, 748, 1024, and 2048, and used the proposed model for training and testing. The recognition performance of different lengths of pulse wave signals is illustrated in Table 4.

**Table 4:** Recognition results of pulse signals with different lengths.

Length	256	748	1024	2048
Accuracy (%)	88.8 ± 0.82	91.3 ± 0.42	90.7 ± 0.34	87.2 ± 0.69
AUC (%)	90.4 ± 1.21	97.4 ± 1.05	92.1 ± 1.16	90.0 ± 1.51

The experimental results revealed that shorter pulse wave signal lengths may lead to insufficient information, affecting the model′s performance. For example, when the waveform length is 256, the accuracy is 88.8%. The classification accuracy is relatively lower, indicating that shorter pulse wave lengths struggle to adequately capture key features, resulting in decreased classification performance. In contrast, longer pulse wave signal lengths, such as 748 and 1024, demonstrated improved classification performance. The model could extract more information from longer time series, enhancing its understanding and feature extraction capabilities of the pulse wave signals, thereby increasing the classification accuracy and overall performance. However, with an increase in pulse wave signal length, computational complexity also correspondingly increases, necessitating a trade‐off between model performance and computational resources.

#### 4.2.2. Learning Rate

**Figure 10:**
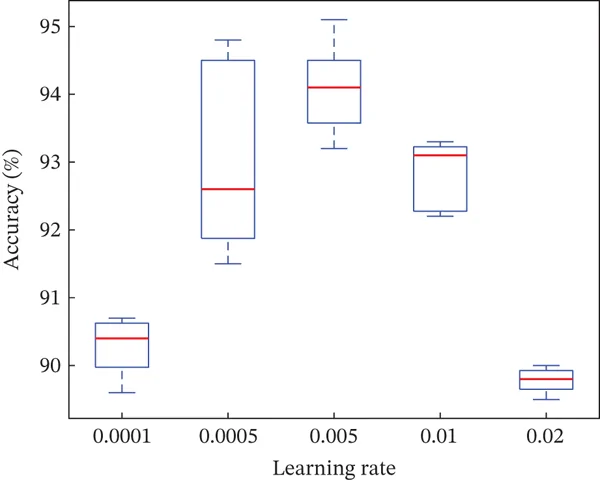
The recognition accuracy under different learning rates.

Selecting a suitable learning rate is crucial for ensuring effective training of our model, as it profoundly impacts the gradient descent process and significantly influences convergence speed and performance. To comprehensively assess its impact, we conducted an analysis across various learning rates: 0.0001, 0.0005, 0.005, 0.01, and 0.02. As depicted in Figure 10, a judicious increase in the learning rate yields a positive influence on model performance. The optimal accuracy is achieved at a learning rate of 0.005, after which further increments lead to a notable accuracy decline. Guided by these findings, we have chosen a learning rate of 0.005 for subsequent phases of the study. This informed decision ensures a balance between enhanced performance and stability in our model′s training process.

#### 4.2.3. Impact of Block A and Block B on Model Performance

This section aims to provide a comprehensive analysis and justification for selecting two Block A and six Block B layers in the model configuration. We first established a baseline model with the initial configuration of two Block A and six Block B, as stated in the manuscript. For each configuration, we measured: (1) the total number of trainable parameters (M); (2) floating‐point operations per forward pass (MFLOPs), calculated using the input length of 748; and (3) average inference latency per sample (ms) measured on our Linux server with a single CPU core to reflect practical deployment conditions. The updated results are summarized in the revised Table 5 (Block A analysis) and Table 6 (Block B analysis).

1. Varying the number of Block A:

**Table 5:** The network performance indicators for Block A.

Block A	Accuracy (%)	Precision (%)	Sensitivity (%)	Specificity (%)	F1	Params (M)	MFLOPs
1	87.1 ± 0.78	85.4 ± 0.71	88.2 ± 0.49	86.2 ± 0.67	0.867	1.24	18.4
2	91.3 ± 0.42	91.7 ± 0.18	91.5 ± 0.23	91.1 ± 0.66	0.916	1.87	26.1
3	91.2 ± 0.91	90.3 ± 0.57	91.6 ± 0.47	90.8 ± 0.29	0.910	2.21	31.8
4	87.5 ± 0.87	89.7 ± 0.31	88.6 ± 0.52	87.1 ± 0.58	0.891	2.58	38.9

**Table 6:** The network performance indicators for Block B.

Block B	Accuracy (%)	Precision (%)	Sensitivity (%)	Specificity (%)	F1	Params (M)	MFLOPs
4	89.2 ± 0.73	88.9 ± 0.55	90.1 ± 0.29	88.4 ± 0.51	0.895	1.42	20.8
6	91.3 ± 0.42	91.7 ± 0.18	91.5 ± 0.33	91.1 ± 0.66	0.916	1.87	26.1
8	90.5 ± 0.84	89.2 ± 0.49	90.6 ± 0.62	89.1 ± 0.39	0.899	2.45	34.2

To rigorously justify the selection of two Block A units, we systematically varied the number of Block A (1, 2, 3, and 4) while holding six Block B fixed, and measured for each configuration: (1) total trainable parameters (M); (2) floating‐point operations per forward pass at input length 748 (MFLOPs); and (3) average per‐sample inference latency (ms) on a single CPU core, reflecting practical edge‐deployment conditions. Results are summarized in the extended Table 5. The two Block A and three Block A configurations yield nearly identical accuracy (91.3% vs. 91.2%) and F1 (0.916 vs. 0.910). However, the three Block A configuration incurs approximately 18% more trainable parameters and approximately 22% higher MFLOPs relative to the two–Block‐A baseline, with a corresponding increase in per‐sample inference latency. Given that the marginal accuracy gain (< 0.1%) falls well within the cross‐validation standard deviation and that the additional computational overhead is nontrivial for real‐time clinical deployment, the two Block A configuration represents the optimal performance–efficiency trade‐off. Configurations with one or four Block A units show substantially lower accuracy (87.1% and 87.5%, respectively), confirming that the dual‐branch structure is necessary for adequate multifrequency feature extraction.

2. Varying the number of Block B

Next, we explored the impact of varying the number of Block B while keeping two Block A units fixed. We tested configurations with four, six (baseline), and eight Block B, respectively. As indicated in Table 6, the experiments demonstrated that the baseline configuration of six Block layers struck a balance between model complexity and performance. Reducing Block B to four units leads to insufficient model capacity (accuracy 89.2%), whereas increasing to eight units adds 31% more parameters without performance improvement (accuracy 90.5%). The six Block B configuration achieves the best accuracy (91.3%) with a favorable efficiency profile. Based on the comprehensive experiments, we reaffirm the selection of two Block A and six Block B as the optimal configuration for our model. The results provide a robust justification for these choices, balancing model performance and computational efficiency for pregnancy pulse identification.

### 4.3. Ablation Experiments Analysis

To comprehensively validate the efficacy of the proposed approach, a thorough analysis was undertaken to assess the impact of distinct model components on the overall model. The focus of this investigation centered on gauging their respective contributions to the classification accuracy. The entire model was dissected into three integral components: the 1DCNN, GRU, and ISENet module. The experimental results are presented in Table 7, encompassing a total of six different combinations.

**Case 1.**

To discern the inherent classification capabilities of the 1DCNN, we isolate this component to gauge its singular impact on accuracy. Operating independently, the 1DCNN achieved an accuracy of 82.3%, showcasing its proficiency in capturing essential features for accurate pulse wave classification.

**Case 2.**

In the standalone GRU scenario, the GRU achieved an accuracy of 85.7%, emphasizing its effectiveness in sequential data analysis for precise pulse wave classification. The GRU′s ability to capture pulse temporal dependencies and long‐range dependencies contributes to its notable accuracy.

**Case 3.**

The hybrid model that combines 1DCNN with GRU achieves an accuracy rate of 88.6%, representing a significant improvement of 6.3% compared with the standalone 1DCNN model. This enhancement highlights the discriminative power that GRU brings to the model.

**Case 4.**

Similarly, the integration of the ISENet module with the 1DCNN yielded an accuracy of 89.9%, highlighting the synergistic effects of feature enhancement and attention mechanisms. The ISENet complements the 1DCNN′s feature extraction capabilities with the weight coefficient, resulting in improved accuracy.

**Case 5.**

The fusion of the GRU with the ISENet further elevated the accuracy to 90.6%. Although its average accuracy is 90.6%, slightly higher than Case 4′s 89.9%, it demonstrates better stability, showcasing the fusion potential of pulse spatiotemporal features based on an attention mechanism.

**Case 6.**

The comprehensive model, integrating all three components, achieved the highest accuracy of 91.3%, underlining the collective strength of feature extraction, sequential analysis, and attentive focus for pulse wave classification. Building on the previous analysis, it can be concluded that all proposed modules affect the classification performance to some extent and among them, ISENet is more important.

**Table 7:** Ablation analysis of the proposed model.

Cases	Component			Accuracy (%)
	1DCNN	GRU	ISENet	
1	√	×	×	82.3 ± 0.84
2	×	√	×	85.7 ± 1.01
3	√	√	×	88.6 ± 0.64
4	√	×	√	89.9 ± 0.82
5	×	√	√	90.6 ± 0.29
6	√	√	√	91.3 ± 0.42

### 4.4. Confusion Matrix and ROC Curve of Proposed Model

**Figure 11:**
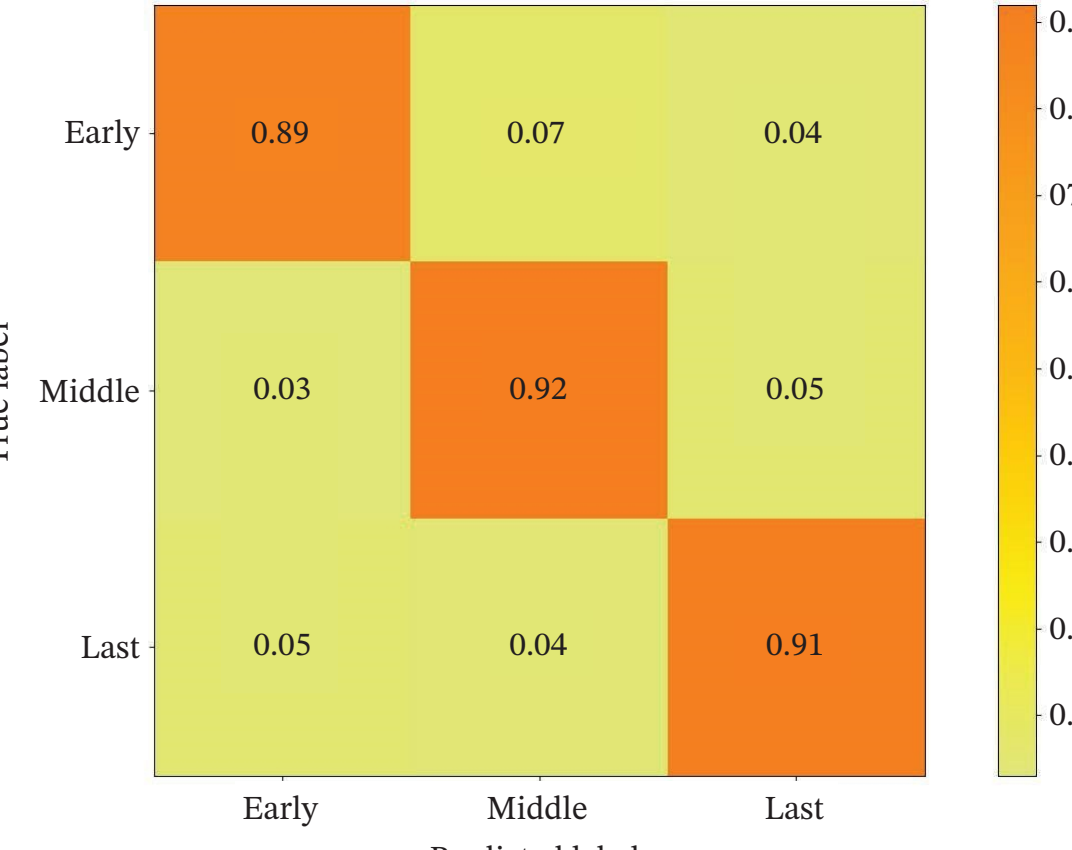
The confusion matrix of proposed model.

**Figure 12:**
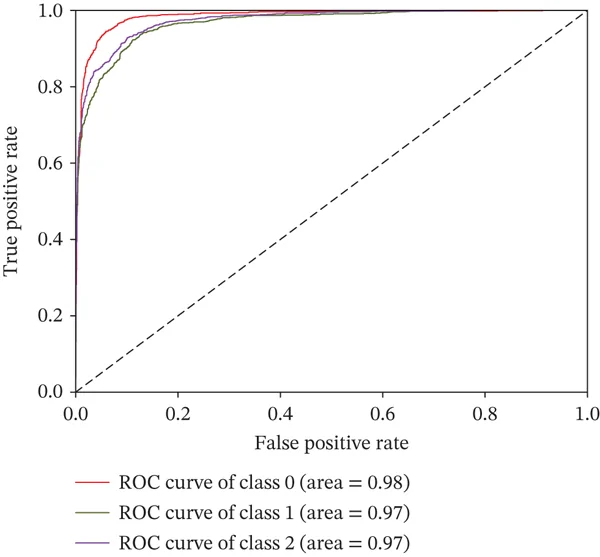
The ROC curve of the proposed model.

In this section, we delve into the assessment of our proposed model′s performance through the utilization of key evaluation metrics, namely the confusion matrix and the ROC curve. The confusion matrix provides a comprehensive overview of the model′s classification results across various classes, whereas the ROC curve offers insights into the model′s ability to balance true positive rates against false positive rates, thereby showcasing its discriminative capacity. Figure 11 presents the confusion matrix for the testing data of pulse. By analyzing Figure 11, the correct prediction rates of the 1DCNN–GRU–ISENet model for the three stages of pregnancy were 89%, 92%, and 91%, respectively. Furthermore, Figure 12 shows the ROC curve of the hybrid model in this paper. The achieved AUC values of 98%, 97%, and 97% for the E, M, and L stages, respectively, underscore the model′s robust discriminative capabilities across different stages of pregnancy.

### 4.5. Comparison With Others Models

**Figure 13:**
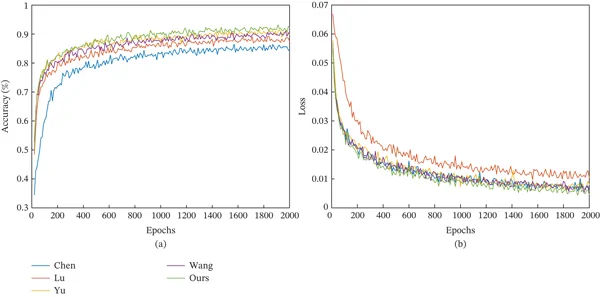
(a,b) Accuracy and loss curves of proposed model against the other works.

To comprehensively assess the efficacy of our proposed method, we meticulously conducted a performance comparison with other approaches within the domain of pulse signal classification. The comparative outcomes of our proposed network against previous counterparts are tabulated in Figure 13. Figure 13 visualizes overall accuracy and loss curves of the proposed model compared with other models. It is evident that the 1DCNN–GRU–ISENet model achieves the fastest convergence and the highest convergence accuracy.

To ensure a fair comparison, we did not directly cite the absolute performance values reported in the original publications. Instead, we reimplemented the core architectures of the comparative methods, including the BP network [16], the SVM–PNN ensemble model [17], the 1DCNN‐GRU network [18], and the LSQ regression model [18]. All models were evaluated under a strictly unified experimental protocol: the identical preprocessed pulse dataset consisting of 184 subjects, the same patient‐wise train/test split of 148 training subjects and 36 test subjects, a consistent data augmentation pipeline, and identical fivefold subject‐wise cross‐validation procedures. All models were trained and evaluated using the same hardware, framework, hyperparameter search protocol, and evaluation metrics.

Under this controlled within‐study comparison, as shown in Table 8, the proposed 1DCNN–GRU–ISENet model achieves the highest accuracy (91.3%) and AUC (97.1%) among all evaluated architectures. The BP network and SVM/PNN attain ACC values of 84.7% and 86.5%, respectively, reflecting limitations in automatic feature learning and temporal modeling capacity. The 1DCNN‐GRU achieves 90.8% ACC and 96.6% AUC, indicating that the addition of ISENet provides a measurable improvement in channel‐wise feature discrimination. The LSQ regression reaches 89.7% ACC and 93.4% AUC, which is lower than the deep learning‐based methods, consistent with the limited representational capacity of handcrafted harmonic features. The observed performance advantage of 1DCNN–GRU–ISENet is attributable to the integration of multiscale local feature extraction (1DCNN), long‐range temporal dependency modeling (GRU), and spatiotemporally aware competitive channel attention (ISENet), which together enable more discriminative representations of stage‐specific pulse waveform patterns.

**Table 8:** Performance comparison with other models.

Study	Models	ACC (%)	AUC (%)
Chen et al. [16]	BP network	84.7	91.8
Lu et al. [17]	SVM/PNN	86.5	94.5
Yu et al. [18]	1DCNN‐GRU	90.8	96.6
Wang et al. [19]	LSQ regression	89.7	93.4
Ours	1DCNN–GRU–ISENet	91.3	97.1

### 4.6. Comparison on Other Benchmark Datasets

To further validate the model, experiments can be conducted on publicly available benchmark datasets related to similar time series data. As shown in Table 9, the results section has been expanded to include a comprehensive comparison with state‐of‐the‐art methods, demonstrating the proposed model′s strong performance by showcasing its effectiveness on other benchmark datasets. In the comparative analysis, our 1DCNN–GRU–ISENet model demonstrates a clear advantage over other studies using the sleep apnea dataset. Specifically, our model achieved an accuracy of 92.1% and a sensitivity of 90.5%. In comparison, although Liu et al.′s CNN transformer model had a slightly higher specificity of 94.1%, our accuracy and sensitivity show a significant improvement, highlighting the effectiveness of our model in detecting sleep apnea. Additionally, although Srivastava et al.′s ALexNet + LSTM model excelled in sensitivity at 95.4%, its accuracy was only 90.9%, indicating a gap compared with our model. Overall, our model strikes a balance between accuracy and sensitivity, showcasing superior detection capability and robustness, making our 1DCNN–GRU–ISENet a more optimal choice.

**Table 9:** Performance comparison on the sleep apnea dataset.

Study	Models	ACC (%)	SEN (%)	SPC (%)
Liu et al. [39]	CNN transformer	88.2	78.5	94.1
Hu et al. [40]	Self‐attention	72.3	80.0	67.6
Yeh et al. [41]	CNN	85.8	80.1	89.4
Shen et al. [42]	MSDA‐1DCNN	89.4	89.8	89.1
Chang et al. [43]	1DCNN	87.9	81.1	92.0
Srivastava et al. [44]	ALexNet + LSTM	90.9	95.4	83.4
Almutairi et al. [45]	CNN LSTM	89.1	89.9	87.8
Rajesh et al. [46]	DWT RF	89.6	85.1	92.8
Ours	1DCNN–GRU–ISENet	92.1	90.5	89.8

### 4.7. Discussion

#### 4.7.1. Dataset Generalizability

We explicitly acknowledge the following limitations regarding dataset scope

1. For dataset generalization limitation: We add federated learning as a future multicenter training scheme in Section 4.7, which can aggregate pulse data from different hospitals, ethnic groups, and regions without uploading sensitive patient pulse signals, solving the data privacy and sample homogeneity problem simultaneously.
2. For clinical error margin: The overall average classification accuracy of the model reaches 91.3%, which meets the demand of preliminary auxiliary screening for TCM pulse diagnosis. We clearly emphasize that the model only provides objective waveform reference information, and the final gestational stage judgment relies on comprehensive clinical diagnosis by TCM physicians.
3. For signal quality control: The SQI module calculates signal smoothness, noise amplitude and waveform integrity of each pulse segment; samples with SQI below the preset threshold will be automatically discarded to avoid misclassification caused by motion artifacts, respiratory interference and poor sensor contact.

#### 4.7.2. Regarding Clinical Workflow

1. Prediction presentation: The model′s output is a probability vector over three pregnancy stages (early, mid, and late). In a clinical decision‐support context, we propose that the system presents the highest probability stage alongside confidence scores for all three classes, enabling the TCM practitioner to exercise clinical judgment in cases of ambiguous predictions.
2. Acceptable error margins: At the current performance level, the system is positioned as an auxiliary decision‐support tool rather than a standalone diagnostic instrument. The model is intended to provide objective supplementary information to aid—not replace—the TCM practitioner′s gestational assessment, consistent with the “auxiliary diagnosis” framing stated in the abstract.
3. Signal quality handling: We have described the signal quality control mechanism used in our preprocessing pipeline, including wavelet‐based noise filtering. We supplement a signal quality index (SQI) detection module in the preprocessing pipeline. Before model inference, SQI will be calculated for each pulse segment; samples below the predefined quality threshold will be marked as invalid and excluded from classification to avoid misdiagnosis caused by motion artifacts, poor sensor contact, and respiratory interference.

## 5. Conclusion

In this study, we developed a hybrid network to recognize pregnancy pulses in three stages. This network integrates parallel stacked branches consisting of 1DCNN blocks, GRU modules, and ISENet modules. One notable advantage of this hybrid model is its ability to classify pregnancy pulse data without the need for manual feature extraction, thereby preserving the richness of information present in the original pulse data. Specifically, the 1DCNN and GRU components are adept at capturing local temporal features and global characteristics of pregnancy pulse patterns, respectively. In addition, the ISENet component dynamically combines spatial relevance and temporal relevance by fusing extracted spatialtemporal features, assigning attention weights to critical spatial features for each pregnancy stage. This approach yielded results, including a classification accuracy of 91.3%, precision of 91.7%, sensitivity of 91.5%, and an F1 score of 0.911.

This study underscores the potential of combining a hybrid deep learning network with TCM diagnosis and treatment, offering innovative avenues that hold significant promise for advancing the clinical development of TCM. In future research, we contemplate introducing classical training models to enhance our proposed model and optimize the identification of key features in pregnancy pulse patterns. In addition, we will collect pulse data from different regions, races, and healthcare institutions to conduct more comprehensive testing in different populations to evaluate its generalization.

## Author Contributions

Nan Li: conceptualization, methodology, formal analysis, writing—original draft. Jiarui Yu: data calculation, resources, writing. Lifeng Dong: visualization, investigation. Yuping Zhao: resources, supervision, funding acquisition.

## Funding

This study was supported by National Key R & D Program of China (No. 2025YFC3512804) and National Key R & D Program of China: Research on Database Construction and Sharing Ecology for Intelligent Research of Traditional Chinese Medicine (AI4TCM) (No. 2025YFC3510900).

## Consent

Informed consent was obtained from all subjects and/or their legal guardians.

## Conflicts of Interest

The authors declare no conflicts of interest.

## Data Availability

The datasets used and/or analyzed during the current study are available from the corresponding authors on reasonable request.
